# Global Phylogenetic Analysis of the CDV Hemagglutinin Gene Reveals Positive Selection on Key Receptor-Binding Sites

**DOI:** 10.3390/v17091149

**Published:** 2025-08-22

**Authors:** Tuba Çiğdem Oğuzoğlu, B. Taylan Koç

**Affiliations:** 1 Department of Virology, Faculty of Veterinary Medicine, Ankara University, 06110 Ankara, Turkey; 2 Department of Virology, Faculty of Veterinary Medicine, Aydın Adnan Menderes University, 09110 Aydın, Turkey

**Keywords:** hemagglutinin, lineage, Nectin-4, SLAM, phylogeny

## Abstract

Canine distemper virus (CDV) is a multi-host morbillivirus whose evolution and host-switching capacity are largely determined by its hemagglutinin (H) gene. To reconsider the molecular evolution of this critical gene, we performed comprehensive phylogenetic, selection, and structural analyses on a curated dataset of 68 representative global H gene sequences. Our phylogenetic reconstruction confirmed the segregation of sequences into distinct, geographically associated lineages. To provide stronger evidence for viral adaptation, we performed a site-specific selection analysis, which identified 15 amino acid sites in the H protein undergoing significant episodic positive selection. Crucially, the majority of the known SLAM and Nectin-4 receptor-binding residues were found to be among these positively selected sites. We further contextualized these findings by mapping the sites onto a 3D homology model of the H protein, which confirmed their location on the exposed surfaces of the receptor-binding domain. This compilation provides quantitative evidence that the key functional regions of the H protein are direct targets for adaptive evolution, which has significant implications for understanding host tropism and the ongoing challenge of vaccine mismatch.

## 1. Introduction

Canine distemper virus (CDV) is a significant pathogen responsible for severe, multisystemic infections in domestic dogs of all ages. This disease typically presents with gastrointestinal and respiratory signs; however, neurological signs can also develop as a distinct clinical manifestation. In adult dogs, a specific neurological syndrome associated with persistent CDV infection is termed “old dog encephalitis” [1,2,3,4].

CDV infects an extensive range of hosts. It extends beyond the dog family (*Canidae*) to include numerous other carnivore families such as bears (*Ursidae*), cats (*Felidae*), weasels (*Mustelidae*), and raccoons (*Procyonidae*), as well as marine mammals like seals (*Phocidae*) and sea lions (*Otariidae*). Notably, the virus has also caused severe disease in non-carnivores, including marmots and specific primates (rhesus and cynomolgus macaques). Extensive research demonstrates that CDV undergoes dynamic genetic processes, which explains its ability to infect an extensive range of hosts [5,6]. In this context, scientific investigations over the last few decades have predominantly focused on characterizing the molecular structure of this virus and tracking its genetic substitutions to better understand its adaptability [7,8,9,10].

CDV is a pathogen classified within the genus *Mobillivirus* of the family *Paramyxoviridae*, and it exhibits a close antigenic relationship and genetic similarity with other prominent members of its genus, including measles virus (MV), rinderpest virus (RPV), and peste des petits ruminants virus (PPRV). The viral genome is composed of a non-segmented, negative-sense, single-stranded ribonucleic acid (RNA) molecule, approximately 15 kb in length [11,12].

The genetic material of CDV directs the synthesis of eight proteins, six of which are structural proteins that each fulfill a particular function in the lifecycle of CDV and its capacity to cause infection. Notably, the nucleocapsid (N), hemagglutinin (H), and fusion (F) proteins, with N being helically structured within the viral envelope, represent key targets for the host’s immune defenses; the H and F proteins are also instrumental in the initial stages of infection, specifically viral attachment to and fusion with host cells [13]. The H and F proteins form the surface spikes of the virion’s envelope, and the H protein mediates binding to host cell receptors like SLAM (CD150) or Nectin 4 (PVRL4) [14].

The H gene is recognized for exhibiting the most significant genetic diversity among the CDV structural genes. Consequently, its protein product, the H protein, shows a level of amino acid variation that surpasses that of any other protein within the *Morbillivirus* genus, potentially accounting for CDV’s broader spectrum of susceptible host species compared to its viral relatives [15]. Alterations within the H gene sequence have been correlated with the virus’s ability to be transmitted between different animal species and with its degree of pathogenicity (Table 1). For example, amino acid substitutions at specific sites within the H protein, such as position 530 and a change from Tyrosine to Histidine at position 549, are associated with enhanced viral virulence [16,17].

This pronounced genetic heterogeneity makes the H gene a preferred marker for the molecular typing of CDV isolates and a valuable tool for examining the evolutionary connections among various strains [21]. CDV is known to form distinct lineages with specific geographic distributions. These lineages are defined and classified based on the phylogenetic clustering of their H gene sequences. The exact number of lineages can vary between studies, partly due to the genetic region analyzed; for instance, studies using the complete H gene have identified eighteen lineages, while other investigations that have focused on partial H gene sequences have documented as many as twenty-two. Although these lineages often show geographical preferences, some degree of co-circulation occurs [12,22]. Recently recognized lineages are typically designated by their geographical point of discovery (continent or subcontinent), often appended with a numerical identifier or a distinctive name like “Rockborn-like” CDV [12]. The array of currently known lineages includes America-1, America-2, North America-3, South America/North America-4, America-5, Canada-1, Canada- 2, Asia-1, Asia-2, Asia-3, Asia-4, Asia-5, Asia-6, Europe Wildlife, Arctic-like, Africa-1, Africa-2, Southern Africa, Europe-1/South America-1, South America-2, South America-3, and Rockborn-like [5,23,24,25,26,27,28]. The characterization and distinction of new lineages rely on variations in the DNA and protein sequences of the H gene. Furthermore, the considerable evolutionary divergence observed in certain CDV lineages from Africa and Asia [29,30] points to genetic drift within the H gene as a driving force for viral evolution and the emergence of new lineages [31]. This continuous accumulation of genetic changes allows the virus to explore new antigenic space and adapt to the diverse receptor landscapes of different carnivore hosts. Understanding this fundamental evolutionary mechanism is therefore critical for tracking the global spread of CDV and anticipating its potential to cross species barriers.

We therefore intended to compile and reanalyze publicly available CDV sequences using recently released bioinformatic tools in this review. Our objective was to reveal the molecular dynamics of certain residues in the H gene by contextualizing the results of phylogenetic and structural analyses.

## 2. Materials and Methods

### 2.1. Metadata Collection and Initial Processing

Complete H gene sequences of CDV were retrieved from the National Center for Biotechnology Information (NCBI) GenBank database. The full list of the 692 accession numbers used as the initial dataset is provided in Appendix A Table A1. Full GenBank records for these accessions were retrieved using NCBI’s Entrez Direct (E-utilities) efetch command.

A custom Bash script was employed to process these records. The primary inclusion criterion was a sequence length between 1700 and 1900 base pairs (bp), and a filter was designed to select complete or near-complete H gene coding sequences while excluding partial fragments.

This process generated an initial FASTA file of filtered H gene sequences and a corresponding metadata file of tab-separated values (TSVs) (Appendix A Table A2).

### 2.2. Representative Sequence Selection

To reduce dataset redundancy and computational load for downstream analyses, a representative set of sequences was selected from the file. This was performed using MMseqs (version 2.0) [32]. Sequences were clustered using the easy-cluster workflow with a sequence identity threshold of 0.98 (98%) and a coverage threshold of 0.8, using coverage mode 0 (coverage of query and target).

The longest sequence from each cluster was typically chosen by MMseqs as the representative, resulting in a FASTA file of 68 representative H gene sequences.

### 2.3. Multiple Sequence Alignment (MSA)

The 68 representative H gene nucleotide sequences were aligned using Clustal Omega (version 1.2) with default parameters [33]. The resulting alignment was saved in FASTA format.

### 2.4. Selection Pressure Analysis

To identify amino acid sites under positive selection, a codon-based analysis was performed on the multiple sequence alignment of the 68 representative H gene nucleotide sequences. We used the Mixed Effects Model of Evolution (MEME) method, implemented on the Datamonkey web server, which is part of the HyPhy suite [34]. MEME is designed to detect sites evolving under episodic positive selection. The analysis was run using the universal genetic code, and sites with a *p*-value ≤0.1 were considered to be under significant diversifying selection.

### 2.5. Homology Modeling and Structural Visualization

To contextualize the locations of positively selected sites, 3D homology models of the H protein were generated for one representative sequence from each of the 14 major lineages identified in our dataset. Protein sequences were submitted to the SWISS-MODEL web server for automated homology modeling [35]. The resulting PDB files were visualized, analyzed, and rendered using UCSF ChimeraX [36]. The mmaker command in ChimeraX was used to superimpose the 14 models to assess structural conservation and variation.

### 2.6. B-Cell Epitope Conservancy Analysis

To computationally assess the potential for antigenic differences between viral lineages, a linear B-cell epitope conservancy analysis was performed. The amino acid sequences of the H protein from representative strains of the vaccine-like America-1 lineage, the Arctic-like lineage, and the European lineage were submitted to the BepiPred-3.0 web server [37]. This tool predicts the location of linear B-cell epitopes based on protein sequences. The resulting epitope score profiles were compared to identify conserved and variable antigenic regions.

### 2.7. Phylogenetic Tree Inference

A Maximum Likelihood (ML) phylogenetic tree was constructed from the aligned representative H gene nucleotide sequences using IQ-TREE (version 3.0.1) [38]. The best-fit nucleotide substitution model was automatically selected by IQ-TREE using ModelFinder Plus (MFP). Branch support was assessed using 1000 replicates of the Ultrafast Bootstrap Approximation (UFBoot). The final ML tree with support values was output in Newick format.

### 2.8. Phylogenetic Network Analysis

To explore potential conflicting phylogenetic signals and reticulate evolutionary patterns, a phylogenetic network was constructed using the Neighbor-Net algorithm, as implemented in SplitsTree6 (version 6.0) [39]. The aligned representative H gene sequences were used as input, and distances were typically calculated using the p-distance method within SplitsTree.

### 2.9. Phylogenetic Tree Visualization and Annotation

The Maximum Likelihood phylogenetic tree was visualized and annotated using iTOL (Interactive Tree Of Life, v6, https://itol.embl.de/ (accessed on 27 May 2025) [40]. The tree was annotated with obtained metadata.

## 3. Results

### 3.1. Phylogenetic Relationships and Lineage Diversity of CDV H Genes

**Figure 1:**
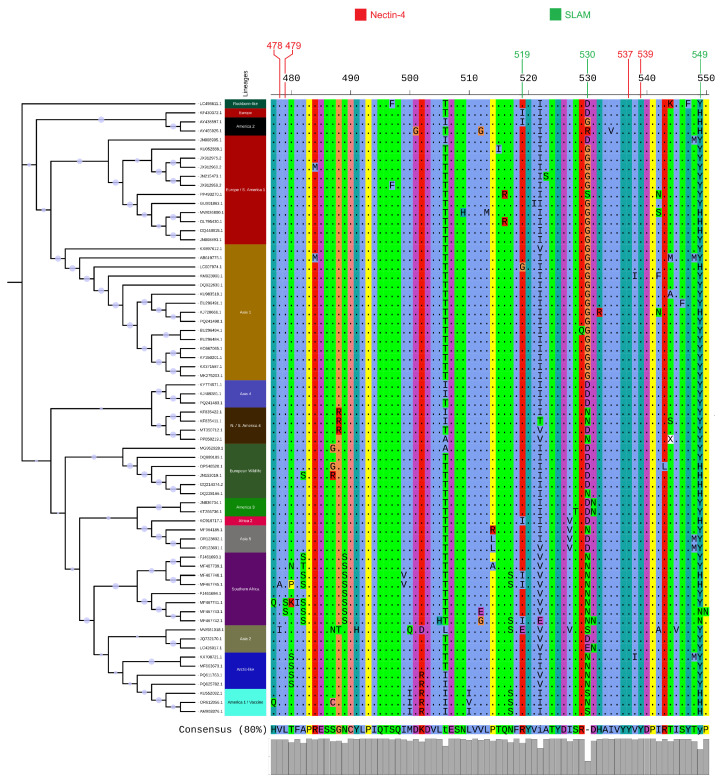
Phylogenetic tree of 68 representative canine distemper virus (CDV) H gene sequences, constructed using the Maximum Likelihood method. The panel on the right shows multiple sequence alignment of the translated H proteins, highlighting key amino acid positions involved in binding to SLAM and Nectin-4 host receptors. Major lineages and variations at these functional sites are visible.

Our initial dataset comprised 692 complete H gene sequences from the GenBank database. Following a redundancy reduction step, a final set of 68 representative sequences was selected for analysis. To elucidate the evolutionary relationships among these sequences, an ML phylogenetic tree was constructed (Figure 1). The analysis revealed a clear and well-supported segregation of the sequences into distinct clades, which correspond to identified CDV lineages. The major clades identified in the dataset included the Europe/South America-1, Asia-1, Southern African, and North/South America-4 lineages, among others. The topology of the tree is characterized by deep, well-supported branches (Ultrafast Bootstrap >95%) that separate the major lineages, which is indicative of substantial genetic divergence between them. Furthermore, the tree highlights the clustering of sequences based on both their geographic origin and, in several instances, their host species, providing a clear map of the global distribution and diversity of these viral lineages.

### 3.2. Variation at Key Receptor-Binding Sites of the Hemagglutinin Protein

To investigate the molecular basis of H gene variability, the translated protein sequences were aligned, and key amino acid positions known to be involved in binding to the SLAM and Nectin-4 host cell receptors were examined (Figure 1). Significant variation was observed at these critical sites across the different lineages. Notably, at position 530, which is a crucial determinant for SLAM receptor interaction, at least five different amino acids (aspartic acid, D; glycine, G; aspargine, N; serine, S; and histidine, H) were observed among the representative strains. Similarly, position 549, another key site for host tropism, showed variation between histidine (H) and tyrosine (Y). Other receptor-binding sites, including positions 478, 519, 528, and 537, also displayed amino acid substitutions between lineages. This molecular diversity at functional receptor-binding sites underscores the strong selective pressures acting on the H protein and provides a potential mechanism for the observed differences in host range and antigenicity among lineages.

### 3.3. Phylogenetic Network Analysis Reveals Conflicting Signals

**Figure 2:**
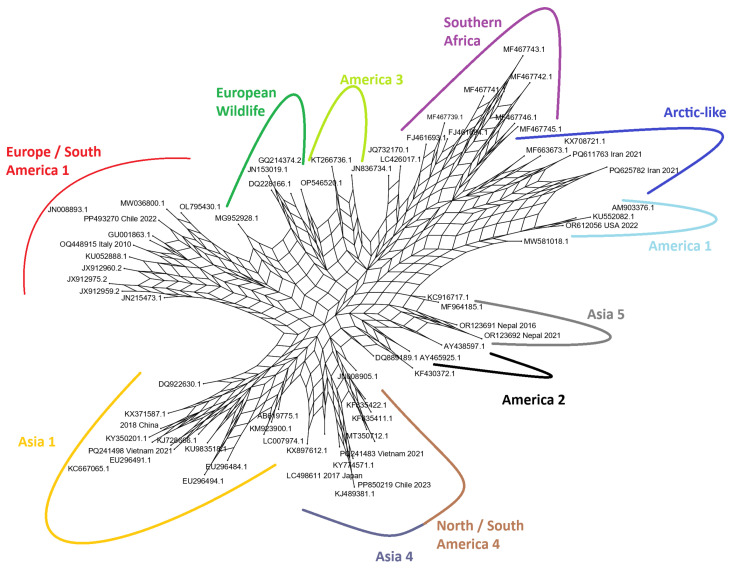
Phylogenetic network of the 68 representative CDV H gene sequences, generated using the Neighbor-Net algorithm in SplitsTree. The network shows major clusters corresponding to the main lineages. The web-like reticulations in the center of the network indicate the presence of conflicting phylogenetic signals within the dataset, highlighting the complex evolutionary history of the H gene.

To explore evolutionary relationships that may not be adequately represented by a strictly bifurcating tree, a phylogenetic network was constructed using the Neighbor-Net algorithm (Figure 2). The resulting network confirmed the major groupings identified in the ML tree, with distinct clusters of related sequences corresponding to the major lineages; however, the network also revealed significant reticulation, visible as dense, web-like structures, particularly in the central part of the network connecting the major lineages. This reticulation indicates the presence of conflicting phylogenetic signals in the dataset. Such signals can arise from processes such as rapid diversification, homoplasy (convergent evolution of amino acid states), or potential recombination events during the evolutionary history of the H gene. The network structure suggests that while the major lineages are clearly distinct, their deep evolutionary relationships are complex and cannot be perfectly resolved into a simple branching tree, highlighting the dynamic and nonlinear nature of CDV H gene evolution.

### 3.4. Identification of Positively Selected Sites in the H Gene

To provide quantitative evidence for adaptive evolution, a selection pressure analysis was performed to identify specific sites in the H gene that are evolving under positive selection. The MEME analysis identified 15 codon sites undergoing significant episodic diversifying selection (*p* value ≤0.1) (Table 2). A considerable finding was that five of these positively selected sites (478, 528, 530, 537, and 549) are known critical residues located within the SLAM and Nectin-4 receptor-binding domains of the H protein. This overlap provides strong evidence that these functionally important regions are key targets of ongoing adaptive evolution.

### 3.5. Structural Mapping of Positively Selected Sites

**Figure 3:**
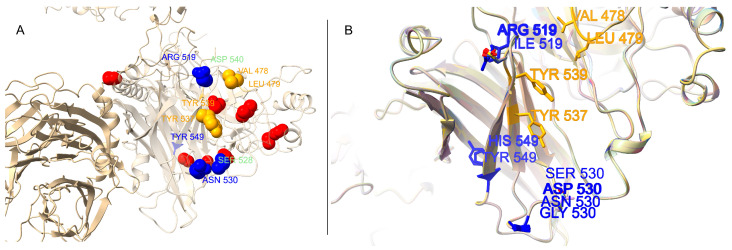
Structural mapping of positively selected sites on the canine distemper virus (CDV) hemagglutinin (H) protein. (**A**) A representative homology model (Arctic-like lineage, PQ611763.1) of the H protein monomer. All 15 sites under positive selection are shown as spheres. Key SLAM receptor-binding sites are highlighted in blue, and Nectin-4 sites are in orange. Positively selected sites with no currently known receptor-binding function are shown in red. (**B**) A superimposed, zoomed-in view of the key receptor-binding region from all 14 modeled lineages, demonstrating the high structural conservation of the protein backbone but variation in the side chains (shown in ball-and-stick representation) at these critical positions.

To visualize the location of the sites under positive selection in a functional context, we generated and analyzed homology models of the H protein. The overall protein fold, which forms a homodimer/tetramer, was found to be highly conserved across all 14 modeled lineages. The 15 positively selected sites were mapped onto the surface of a representative H protein monomer (Figure 3). The visualization confirms that these sites, particularly the critical receptor-binding residues, are located on the exposed outer surfaces of the protein’s head domain. A superimposed view of the key receptor-binding sites across all 14 lineage models reveals subtle but potentially significant differences in the size, charge, and orientation of amino acid side chains at these functionally critical positions (Figure 3B), providing a structural basis for potential differences in receptor interactions.

### 3.6. Predicted Antigenic Differences Between Vaccine and Field Strains

**Figure 4:**
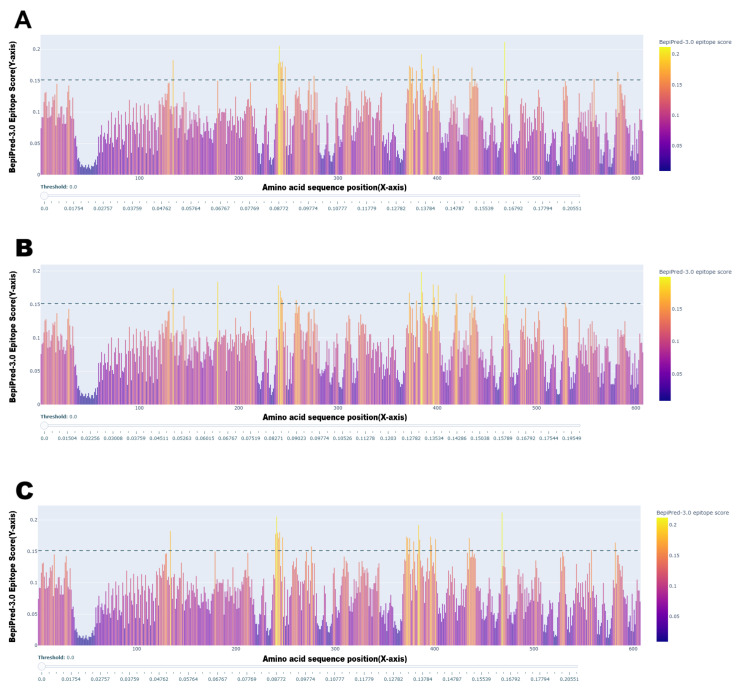
Comparative B-cell epitope prediction for the CDV H protein. The plots show the predicted linear B-cell epitope scores (Y-axis) along the amino acid sequence position (X-axis) for representative sequences from (**A**) the vaccine-like America-1 lineage, (**B**) the Arctic-like lineage, and (**C**) the European lineage. Peaks above the dashed threshold line (score>0.15), which is the default cutoff recommended by the BepiPred-3.0 tool for identifying likely epitopes, indicate predicted antigenic regions. This epitope conservation analysis reveals both conserved and variable antigenic regions between the vaccine and field strains.

To investigate the molecular basis for potential vaccine mismatch, we compared the predicted B-cell epitope profiles of the H protein from the vaccine-like America-1 lineage with two representative field strains (Arctic-like and European lineages) (Figure 4). While the overall antigenic profiles showed broad conservation across the protein, several regions exhibited notable differences in their predicted epitope scores. For instance, a region around site 400 showed a significantly higher predicted antigenicity in the field strains compared to the vaccine strain. Furthermore, subtle variations in epitope scores were observed in the functionally critical receptor-binding region (sites 530–550). The overlap of these variable antigenic regions with sites found to be under positive selection suggests that evolutionary pressure is actively shaping the antigenic surface of the H protein, providing a potential mechanism for immune evasion in vaccinated populations.

## 4. Discussion

This comprehensive bioinformatic analysis of 68 representative CDV H gene sequences provides a clear snapshot of the virus’s ongoing global evolution and highlights several key themes critical for veterinary medicine and wildlife conservation. Our findings, derived from both Maximum Likelihood phylogeny and phylogenetic network analysis, reveal the extensive genetic diversity of CDV and underscore the complex interplay between viral lineage, geographic distribution, and host range.

### 4.1. Global Dispersal and Co-Circulation of CDV Lineages

The phylogenetic tree (Figure 1) and the Neighbor-Net analysis (Figure 2) both successfully segregated the representative strains into distinct, well-supported clades corresponding to established CDV lineages. Our analysis visualizes the cosmopolitan nature of certain lineages, such as Europe/South America-1, which was identified in samples from South America (Brazil, Chile, Uruguay) and Europe (Italy, Germany). This broad distribution supports the hypothesis of frequent inter-continental viral traffic, likely facilitated by the global movement of domestic dogs [41,42,43]. In contrast, other lineages like Southern African appear more geographically constrained, though the presence of multiple sub-clades within this lineage in a single country (South Africa) points to complex local evolution and circulation among a diverse array of wildlife hosts, including lions (*Panthera leo*), spotted hyenas (*Crocuta crocuta*), and African wild dogs (*Lycaon pictus*) [44,45,46]. A significant finding is the co-circulation of multiple distinct lineages within the same geographic region. For instance, Italy was found to harbor strains from the Arctic-like, European Wildlife, and Europe/South America-1 lineages. This demonstrates that viral populations in a given area are not static but are shaped by repeated, independent introduction events, likely from different sources or hosts. The Neighbor-Net analysis (Figure 2) further supports this complexity, where the dense, web-like reticulations connecting the major lineage clusters suggest a history of rapid diversification and potentially conflicting phylogenetic signals that a simple tree cannot fully capture [45,47,48,49].

### 4.2. The Wildlife–Domestic Interface, Host Adaptation, and H Gene’s Role

A central theme emerging from our results is the critical role of the H gene in host adaptation, particularly at the wildlife–domestic animal interface. The data clearly show that major lineages are not restricted to domestic dogs. The Southern African lineage was found in a variety of wild carnivores [45], the European Wildlife lineage was identified in foxes (*Vulpes vulpes*) and martens (*Martes martes*) [50,51], and the Asia-1 lineage, while common in dogs, was also found in a raccoon dog (*Nyctereutes procyonoides*) and a rhesus macaque (*Macaca mulatta*) [20,23,44,52,53]. This supports the hypothesis that wild carnivores act as significant wildlife hosts and potential reservoirs, maintaining viral diversity and facilitating spillover events into domestic dog populations and across different wildlife species. The molecular basis for this host plasticity is likely rooted in the high variability of the H gene, particularly at receptor-binding sites. Our alignment analysis (Figure 1) highlights extensive amino acid substitutions at key positions like 530 and 549, which are known to mediate binding to the SLAM and Nectin-4 receptors and are critical for determining host tropism. The diversity at these sites (e.g., at least five different amino acids at position 530) across lineages likely reflects ongoing adaptation to the specific receptor variants of different host species [19,21,25,44,54]. This continuous evolution allows the virus to explore new ecological niches, posing a persistent threat to a wide range of carnivores, including endangered species like the Iberian lynx (*Lynx pardinus*) and Amur tiger (*Panthera tigris*) [55,56]. This dynamic highlights the potential for transmission in both directions, from domestic dogs to wildlife (spillover) and from wildlife reservoirs back to dogs (spillback), which complicates disease control efforts. Thus, high vaccination coverage in domestic dogs is crucial, and strategies such as wildlife vaccination or vaccinated dog buffer zones around conservation areas should be considered to mitigate spillover risks.

The domains on the H gene that bind to the SLAM and Nectin-4 receptors act as the key factors driving host and lineage shifts and are illustrated in Figure 1. Specifically, these regions involve amino acids at positions 519, 530, and 549 for SLAM binding and at 478, 479, 537, and 539 for Nectin-4 binding. While recent research has identified substitutions at other loci, the significance of these aforementioned regions remains undiminished. Notably, Figure 1 and Table 2 indicate a higher frequency of substitutions within the SLAM-binding amino acids. A plausible explanation for this observation is the role of SLAM as a cellular entry receptor, which would impose a more intense selective pressure on the virus to adapt [57]. In addition to host switching, it is also most likely to emerge as the most significant factor influencing lineage differentiation. Our findings of positive selection on the H gene align with known polymorphisms in carnivore SLAM receptors. Residue 530 (glycine in canids; tyrosine or histidine in felids or mustelids) and residue 549 strongly influence receptor-binding specificity. Positive selection at these sites provides evolutionary evidence of CDV adaptation to host receptor diversity, highlighting the molecular basis of its host-switching capacity [43,58,59].

Our structural modeling also contextualizes these findings. The 3D model (Figure 3) indicates that these positively selected sites are located on the exposed outer surfaces of the protein head domain, consistent with their role in host–receptor interaction and potential immune evasion. Furthermore, the comparative modeling of 14 distinct lineages (Figure 3B) revealed that while the overall protein fold is highly conserved, the specific amino acid substitutions at these key sites alter the local size, charge, and chemistry of the receptor-binding interface. For example, the variation at site 530 between a small glycine (common in vaccine-like strains) and a larger, charged aspartic acid (common in Asia-1) provides a structural hypothesis for how different lineages adapt to the specific receptor variants of different host species [19].

### 4.3. Implications for Vaccine Efficacy

A crucial point of discussion arising from our phylogenetic analysis is the significant genetic divergence between currently circulating field strains and the vaccine strains. The vast majority of commercial CDV vaccines are derived from the historical America-1 lineage (e.g., Onderstepoort strain) [24,45]. Our results show that the representative strains from major circulating lineages like Asia-1, Europe/South America-1, and Southern African form distinct clades that are phylogenetically distant from the America-1 lineage. This genetic gap, particularly in the highly antigenic H gene, is a leading hypothesis for observed vaccine failures and outbreaks in vaccinated dog populations [44,60].

B-cell epitope conservancy analysis (Figure 4) also provides computational evidence to support this “vaccine mismatch” hypothesis. The analysis revealed notable differences in the predicted epitope profiles between the vaccine strain and the representative field strains (Arctic-like and Europe), which have been substantially detected throughout Europe [61,62]. The location of these antigenic differences within regions under positive selection suggests that the virus is actively evolving to evade the host immune response [2,10,25].

Our analysis revealed several substitutions in critical receptor-binding residues of the H protein, particularly at positions 530 and 549, that diverge significantly from those in the America-1 vaccine strain. Due to their critical role in SLAM receptor engagement and their location within positively selected B-cell epitope regions [17,19,63,64], these changes could reduce vaccine-induced protection and enable cross-species transmission [24,44]. Structural modeling further indicated that variation in side-chain properties at these sites may alter receptor-binding interfaces [8,16]. This factor could contribute to the emergence of immune-evading variants in regions with co-circulating divergent lineages, such as Europe and Asia [24,31]. These findings underscore the urgent necessity for vaccines that incorporate antigens from contemporary field strains, as well as the need for enhanced genomic surveillance to detect variants with reduced neutralization potential.

Our study has a limitation in that the dataset of 68 representative sequences might not be sufficient to reliably estimate substitution rates or root-to-tip divergence. Larger datasets could yield more accurate results. Nevertheless, our findings encourage future studies to conduct these analyses.

## 5. Conclusions

Extensive research has established the importance of CDV and its geographical distribution patterns. The considerable diversity observed among CDV lineages is primarily attributed to their specific geographical origins.

The clear phylogenetic and geographic structuring of lineages, coupled with extensive host switching and significant molecular variation at key functional sites, highlights the complex challenges in controlling canine distemper. The deep genetic divergence between circulating field strains and current vaccine lineages is a point of major concern that warrants further investigation and may necessitate a re-evaluation of global vaccination strategies. A “One Health” approach, integrating surveillance of domestic dogs, wildlife, and the environment, is essential to effectively mitigate the threat posed by this formidable multi-host virus.

Moving forward, effective control of CDV will require proactive measures that extend beyond current vaccination practices. We recommend the development of regionalized or updated vaccine formulations that incorporate antigens from prevalent local lineages, particularly in regions where multiple divergent strains co-circulate. Expanded genomic surveillance is urgently needed in underrepresented areas such as Africa and Southeast Asia to identify emerging variants before they spread widely. Finally, experimental validation of our structural predictions, including receptor-binding and neutralization assays, will be essential to confirm the functional consequences of the observed amino acid substitutions and to guide evidence-based vaccine updates.

## Figures and Tables

**Table 1:** Key amino acid residues in the canine distemper virus (CDV) hemagglutinin (H) protein involved in receptor binding.

Residue	Associated	Reported Functional Effect	Key
Position	Receptor		References
478	Nectin-4	Critical for Nectin-4 binding and viral entry into epithelial cells.	[18]
479	Nectin-4	Contributes to Nectin-4 interaction.	[18]
519	SLAM	Part of the SLAM binding interface.	[19]
530	SLAM	Major determinant of host range and SLAM binding affinity.	[19]
537	Nectin-4	Critical for Nectin-4 binding; substitutions can affect viral entry.	[18,19]
539	Nectin-4	Part of the Nectin-4 binding interface.	[18,19]
549	SLAM	Key determinant of host tropism and SLAM binding efficiency.	[8,19,20]

**Table 2:** Amino acid sites under episodic positive selection in the CDV H gene, as identified by MEME analysis (*p* ≤0.1). Sites with known receptor-binding functions are highlighted in bold.

Codon Site	*p*-Value (MEME)	Known Function/Location
28	0.088	-
70	0.068	-
169	0.054	-
280	0.024	-
298	0.088	-
300	0.015	-
395	0.054	-
451	0.088	-
460	0.054	-
**478**	**0.054**	**Nectin-4 Receptor Binding**
**528**	**0.001**	**SLAM Receptor Binding**
**530**	**<0.001**	**SLAM Receptor Binding**
**537**	**0.015**	**Nectin-4 Receptor Binding**
**540**	**0.054**	**SLAM Receptor Binding**
**549**	**<0.001**	**SLAM Receptor Binding**

## Data Availability

The data presented in the study were obtained from GenBank, and further inquiries can be directed to the corresponding author.

## Appendix A. Supplementary Metadata

**Table A1:** List of 692 GenBank accession numbers for the complete H gene sequences of canine distemper virus (CDV) used as the initial dataset for this study.

GenBank Accession Numbers			
PQ625782.1	PQ611763.1	PQ241483.1	PQ241484.1
PQ241485.1	PQ241486.1	PQ241487.1	PQ241488.1
PQ241489.1	PQ241490.1	PQ241491.1	PQ241492.1
PQ241493.1	PQ241494.1	PQ241495.1	PQ241496.1
PQ241497.1	PQ241498.1	PQ241499.1	PQ241500.1
PQ241501.1	PQ241502.1	PP977570.1	PP894823.1
PP850218.1	PP850219.1	PP850220.1	PP850221.1
PP493270.1	PP493271.1	PP493272.1	PP493273.1
PP316238.1	PP316239.1	OR612054.1	OR612056.1
OR243301.1	OR123691.1	OR123692.1	OQ448914.1
OQ448915.1	OP546520.1	OP546521.1	OP546522.1
OP546523.1	OP546524.1	OP546525.1	OP546526.1
OP546527.1	OP546528.1	OP546529.1	ON458033.1
OL795421.1	OL795422.1	OL795423.1	OL795424.1
OL795425.1	OL795426.1	OL795427.1	OL795428.1
OL795429.1	OL795430.1	ON929298.1	ON929300.1
ON093253.1	MW581018.1	MW581019.1	MW581020.1
MW581021.1	MW581022.1	MW581023.1	MW581024.1
MW581025.1	MW581026.1	MW015089.1	MW015090.1
MT350712.1	MT350713.1	MT350714.1	MT350715.1
MT350716.1	MT350717.1	MW036777.1	MW036781.1
MW036782.1	MW036787.1	MW036798.1	MW036800.1
MW036808.1	MW036817.1	MW036820.1	MT292106.1
MN044670.1	MN044671.1	MN044672.1	MN044673.1
MN044674.1	MN044675.1	MN044676.1	MN044679.1
MN044681.1	MN044684.1	MN044685.1	MN044686.1
MN044687.1	MN044688.1	MN044693.1	MN044694.1
MN044698.1	MN044699.1	MN044700.1	MT066171.1
LC498611.1	MK275197.1	MK275198.1	MK275199.1
MK275200.1	MK275201.1	MK275202.1	MK275203.1
MK275204.1	MK689164.1	MK689165.1	MK689166.1
MG952927.1	MG952928.1	MG952929.1	LC426017.1
MF663673.1	MF663674.1	MG922458.1	MG922459.1
MG922460.1	MF467738.1	MF467739.1	MF467740.1
MF467741.1	MF467742.1	MF467743.1	MF467744.1
MF467745.1	MF467746.1	MF467747.1	KX708721.1
KX708722.1	KX708723.1	KX708724.1	KX708725.1
KX708726.1	KX708727.1	KX708728.1	KX708729.1
KX708730.1	KX708731.1	KX708732.1	MF926598.1
MF926605.1	MF926606.1	MF926607.1	MF926608.1
MF926609.1	MF926610.1	MF926611.1	MG827088.1
MG827089.1	MG827090.1	KY774571.1	KY774572.1
KY774573.1	KY774574.1	KY774575.1	KY774576.1
KY774577.1	KY774578.1	KY350199.1	KY350200.1
KY350201.1	KY350202.1	KY350203.1	KY350204.1
KY350205.1	KY350206.1	KY350207.1	KY350208.1
KY350209.1	KY350210.1	KY350211.1	KY350212.1
KY350213.1	KX897609.1	KX897610.1	KX897611.1
KX897612.1	MF964178.1	MF964179.1	MF964180.1
MF964181.1	MF964182.1	MF964183.1	MF964184.1
MF964185.1	MF964186.1	MF964187.1	MF964190.1
KX943319.1	KX943320.1	KX943321.1	KX943322.1
KX943323.1	KX943324.1	KX943325.1	KX943326.1
MF100798.1	MF100799.1	MF100800.1	MF100801.1
MF100802.1	MF100803.1	MF100804.1	MF100805.1
MF100806.1	MF100807.1	MF100808.1	MF100809.1
MF100810.1	MF100811.1	KU052888.1	KU052889.1
KU052890.1	KU052891.1	KU052892.1	KU052893.1
KU052894.1	KU052895.1	KU052896.1	KU052897.1
KX371584.1	KX371585.1	KX371586.1	KX371587.1
KX371588.1	KU342688.1	KU342692.1	LC159583.1
LC159584.1	LC159585.1	LC159586.1	KU847961.1
KU552082.1	KU521343.1	KU521344.1	KU521345.1
KU521346.1	KU983518.1	KU983519.1	KU983520.1
KU983521.1	KU983522.1	KU983523.1	KU030831.1
KU030832.1	KU030833.1	KT266736.1	KT119347.1
KR185351.1	KT341044.1	KT341045.1	KT341046.1
KT341047.1	KT341048.1	KP872502.1	KR002657.1
KR002658.1	KR002659.1	KR002660.1	KR002661.1
KR074995.1	LC007974.1	LC007975.1	LC007976.1
KP127955.1	KP127956.1	KP127957.1	KP127958.1
KP127959.1	KP127960.1	KP127961.1	KP127962.1
KP127963.1	KP127964.1	KP127965.1	KP127966.1
KP127967.1	KM923900.1	KJ600773.1	KJ600774.1
KJ600775.1	KJ600776.1	KM386683.1	KM115532.1
KM115533.1	KM115534.1	KM115535.1	KM115536.1
KC916714.1	KC916715.1	KC916716.1	KC916717.1
KJ865300.1	KJ728666.1	KJ728667.1	KJ685550.1
KF835411.1	KF835412.1	KF835413.1	KF835414.1
KF835415.1	KF835416.1	KF835417.1	KF835418.1
KF835419.1	KF835420.1	KF835421.1	KF835422.1
KF835423.1	KF835424.1	KF835425.1	KJ579444.1
KJ160517.1	KJ437593.1	KJ437594.1	KJ437595.1
KJ437596.1	KJ489381.1	KJ489382.1	KJ489383.1
KF430337.1	KF430338.1	KF430339.1	KF430340.1
KF430341.1	KF430342.1	KF430343.1	KF430344.1
KF430345.1	KF430346.1	KF430347.1	KF430348.1
KF430349.1	KF430350.1	KF430351.1	KF430352.1
KF430353.1	KF430354.1	KF430355.1	KF430356.1
KF430357.1	KF430358.1	KF430359.1	KF430360.1
KF430361.1	KF430362.1	KF430363.1	KF430364.1
KF430365.1	KF430366.1	KF430367.1	KF430368.1
KF430369.1	KF430370.1	KF430371.1	KF430372.1
KF430373.1	KF430374.1	KF430375.1	KF430376.1
KF430377.1	KF880677.1	KF880678.1	KF880679.1
KF880680.1	KF880681.1	KF880682.1	KF880683.1
KF880684.1	KF767692.1	JX844218.1	JX844219.1
JX844220.1	JX844221.1	KF006819.1	KF006820.1
KC966928.1	KC966929.1	JQ319389.1	JQ319391.1
JQ319392.1	JQ319393.1	JQ319395.1	JQ319396.1
JQ319397.1	JQ319398.1	JQ319399.1	JQ327707.1
JQ327708.1	JN008893.1	JN008894.1	JN008895.1
JN008896.1	JN008897.1	JN008898.1	JN008899.1
JN008900.1	JN008901.1	JN008902.1	JN008903.1
JN008904.1	JN008905.1	JN008906.1	JN008907.1
KC667065.1	KC667067.1	KC590510.1	KC494688.1
AB475098.1	KC257463.1	KC257464.1	JQ966309.1
JX912958.2	JX912959.2	JX912960.2	JX912961.2
JX912963.2	JX912964.2	JX912965.2	JX912966.2
JX912968.2	JX912971.2	JX912975.2	JX912976.2
JX912978.2	JF810106.1	JF810107.1	JF810108.1
JF810109.1	JF810110.1	JF810111.1	JN812975.1
JN812976.1	JX276746.1	JQ732170.1	JQ732171.1
JQ732172.1	JQ732173.1	JQ732174.1	JQ732175.1
JQ732176.1	JQ732177.1	JN836734.1	JN836735.1
JN836736.1	JN836737.1	JN622007.1	JN381191.1
JN215473.1	JN215474.1	JN215475.1	JN215476.1
JN215477.1	AB619774.1	AB619775.1	JF343962.1
JN153019.1	JN153020.1	JN153021.1	JN153022.1
JN153023.1	JN153024.1	JN153025.1	AB605890.1
AB605891.1	HQ657209.1	HQ850147.1	HM443704.1
HM443706.1	HM443707.1	HM443708.1	HM443709.1
HM443710.1	HM443711.1	HM443712.1	HM443714.1
HM443715.1	HM443716.1	HM443717.1	HM443718.1
HM443719.1	HM443720.1	HM443721.1	HM443722.1
HM443723.1	HM443724.1	HM443725.1	HM443726.1
HQ007902.1	GU810819.1	HQ128599.1	HQ128600.1
HQ128601.1	AB606410.1	HM623891.1	HM623893.1
HM623895.1	HQ403645.1	HM448829.1	HM448830.1
HM448831.1	HM448832.1	HM448833.1	HM448834.1
HM749644.1	GU001863.1	GU001864.1	HM120874.1
HM165273.1	FJ461693.1	FJ461694.1	FJ461695.1
FJ461696.1	FJ461697.1	FJ461698.1	FJ461699.1
FJ461700.1	FJ461701.1	FJ461702.1	FJ461703.1
FJ461704.1	FJ461705.1	FJ461706.1	FJ461707.1
FJ461708.1	FJ461709.1	FJ461710.1	FJ461711.1
FJ461712.1	FJ461713.1	FJ461714.1	FJ461715.1
FJ461716.1	FJ461717.1	FJ461718.1	FJ461719.1
FJ461720.1	FJ461721.1	FJ461722.1	FJ461723.1
FJ461724.1	GQ332530.1	GQ332531.1	GQ332532.1
GQ332533.1	GQ332534.1	GQ332535.1	GQ214369.2
GQ214373.2	GQ214374.2	GQ214376.2	GQ214378.2
GQ214380.2	GQ214384.2	FJ848530.1	FJ848531.1
FJ848532.1	FJ848533.1	FJ848534.1	FJ848535.1
FJ848536.1	FJ851450.1	FJ851451.1	FJ851452.1
FJ851453.1	FJ851454.1	FJ851455.1	FJ851456.1
FJ851457.1	FJ851458.1	FJ810213.1	FJ810214.1
FJ810215.1	FJ694852.1	FJ694853.1	FJ705230.1
FJ705231.1	FJ705232.1	FJ705233.1	FJ705234.1
FJ705235.1	FJ705236.1	FJ705237.1	FJ705238.1
FJ705239.1	FJ605455.1	FJ535063.1	EU934233.1
EU296481.1	EU296482.1	EU296483.1	EU296484.1
EU296485.1	EU296486.1	EU296487.1	EU296488.1
EU296489.1	EU296490.1	EU296491.1	EU296492.1
EU296493.1	EU296494.1	FJ423604.1	FJ423605.1
FJ423606.1	FJ423607.1	FJ423608.1	FJ423609.1
FJ392651.1	FJ392652.1	FJ409464.1	FJ405223.1
FJ405224.1	EU716072.1	EU716073.1	EU716074.1
EU716075.1	FJ011004.1	FJ011005.1	FJ040791.1
EU684265.1	EU564812.1	EU564813.1	EU379560.1
EU325720.1	EU325721.1	EU325722.1	EU325723.1
EU325724.1	EU325725.1	EU325726.1	EU325727.1
EU325728.1	EU325729.1	EU325730.1	EU325731.1
EU252148.1	EU252149.1	AM903376.1	EU143737.1
DQ889177.1	DQ889178.1	DQ889179.1	DQ889180.1
DQ889181.1	DQ889182.1	DQ889183.1	DQ889184.1
DQ889185.1	DQ889186.1	DQ889187.1	DQ889188.1
DQ889189.1	DQ903854.1	EF445051.1	EF445052.1
EF445053.1	EF445054.1	EF042818.1	DQ922630.1
DQ226087.1	DQ226088.1	DQ228166.1	DQ494317.1
DQ494318.1	DQ494319.1	DQ191175.1	AY548109.1
AY548110.1	AY548111.1	AY498692.1	AY465925.1
AY438597.1	AB040766.1	AB040767.1	AB040768.1

**Table A2:** Metadata for the 68 representative canine distemper virus (CDV) H gene sequences used in the phylogenetic and network analyses. The table includes GenBank accession number, collection year, country of origin, host species, and assigned lineage.

Accession	Year	Country	Host	Lineage
LC498611.1	2017	Japan	Vulpes zerda	Rockborn-like
MK275203.1	2018	China	Canis sp.	Asia-1
MG952928.1	2014	Argentina	Canis lupus familiaris	European Wildlife
LC426017.1	2018	Japan	Canis lupus familiaris	Asia-2
MF663673.1	2016	Italy	Dog	Arctic-like
MF467739.1	2016	South Africa	African wild dog	Southern African
MF467741.1	2016	South Africa	African wild dog	Southern African
MF467742.1	2016	South Africa	African wild dog	Southern African
MF467743.1	2016	South Africa	African wild dog	Southern African
MF467745.1	2015	South Africa	Lion	Southern African
MF467746.1	2015	South Africa	Lion	Southern African
KX708721.1	2013	Russia	Martes zibellina	Arctic-like
KY774571.1	2016	China	Dog	Asia-4
KY350201.1	2016	China	Dog	Asia-1
KX897612.1	2015	China	Tiger	Asia-1
MF964185.1	2016	India	Dog	Asia-5
KU052888.1	2014	Chile	Dog	Europe/South America-1
KX371587.1	2016	China	Dog	Asia-1
KU552082.1	1982	China	Dog	Arctic-like
KU983518.1	2014	China	Dog	Asia-1
KT266736.1	2014	Mexico	Canine	America-3
LC007974.1	2010	Japan	Vulpes vulpes	Asia-1
KM923900.1	2008	China	Monkey	Asia-1
JN008893.1	2011	Greece	Dog	Europe/South America-1
JN008905.1	2011	Greece	Vulpes vulpes	Europe/South America-1
KC667065.1	2012	China	Dog	Asia-1
JX912959.2	2012	Brazil	Dog	Europe/South America-1
JX912960.2	2012	Brazil	Dog	Europe/South America-1
JX912975.2	2012	Brazil	Dog	Europe/South America-1
JQ732170.1	2012	Brazil	Dog	Europe/South America-1
JN836734.1	2009	USA	Martes pennanti	America-3
JN215473.1	2007	Uruguay	Dog	Europe/South America-1
AB619775.1	2009	Japan	Racoon Dog	Asia-1
JN153019.1	2007	Germany	Racoon	European Wildlife
EU296484.1	2006	Taiwan	Canine	Asia-1
EU296491.1	2006	Taiwan	Canine	Asia-1
EU296494.1	2007	Taiwan	Canine	Asia-1
AM903376.1	2006	India	Dog	America-1
DQ889189.1	2006	Hungary	Stone marten	European Wildlife
DQ922630.1	2006	China	Dog	Asia-1
DQ228166.1	2006	Hungary	Dog	European Wildlife
AY465925.1	2004	USA	Raccoon	America-2
AY438597.1	2004	USA	Dog	America-2
PQ625782.1	2021	Iran	Dog	Arctic-like
PQ611763.1	2021	Iran	Dog	Arctic-like
PQ241483.1	2023	Vietnam	Dog	Asia-4
PQ241498.1	2018	Vietnam	Dog	Asia-1
PP850219.1	2023	Chile	Dog	North/South America-4
PP493270.1	2022	Chile	Dog	Europe/South America-1
OR612056.1	2022	USA	Domestic Ferret	Arctic-like
OR123691.1	2016	Nepal	Panthera pardus	Asia-5
OR123692.1	2021	Nepal	Panthera pardus	Asia-5
OQ448915.1	2010	Italy	Ferret	Europe/South America-1
OP546520.1	2021	Italy	Fox	European Wildlife
OL795430.1	2016	Germany	Red fox	Europe/South America-1
MW581018.1	2015	USA	Grey seal	Asia-2
MT350712.1	2018	Peru	Canis lupus familiaris	North/South America-4
MW036800.1	2019	Italy	Fox	Europe/South America-1
KC916717.1	1994	Tanzania	Spotted hyena	Africa-2
KJ728666.1	2013	China	Raccoon dog	Asia-1
KF835411.1	2011	Colombia	Dog	North/South America-4
KF835422.1	2012	Colombia	Dog	North/South America-4
KJ489381.1	2012	China	Dog	Asia-4
KF430372.1	2007	Denmark	Neovison vison	Europe
GU001863.1	2005	Spain	Lynx pardinus	Europe/South America-1
FJ461693.1	2007	South Africa	Dog	Southern African
FJ461694.1	2007	South Africa	Dog	Southern African
GQ214374.2	2006	Austria	Badger	European Wildlife
